# Protein Language Model Embeddings Reveal Proteome-Scale Ortholog Divergence Relevant to Cross-Species Pharmacology

**DOI:** 10.3390/proteomes14030036

**Published:** 2026-07-23

**Authors:** Taichi Endoh, Gerry Amor Camer, Kotetsu Kayama, Daiji Endoh, Hiroki Teraoka

**Affiliations:** 1 MATO Corporation, Exis Building 4F, 5-26-22 Higashi-Ohi, Shinagawa-ku, Tokyo 140-0011, Japan; takkunn1155@gmail.com; 2 School of Veterinary Medicine, Rakuno Gakuen University, Ebetsu 069-8501, Japan; gercamer@uep.edu.ph (G.A.C.); choli666@gmail.com (K.K.); 3 College of Veterinary Medicine, University of Eastern Philippines, Catarman 6400, Philippines; 4 College of Science, Polytechnic University of the Philippines, Manila 1016, Philippines; 5 Medical Corporation Tetsuryukai, Kasai Home Care Clinic, 101, 18-12 Nakakasai 8-chome, Edogawa-ku, Tokyo 134-0083, Japan; 6 Knowledge Connect, K2 SQUARE, 7F, 12-5 Minami 2-jo Nishi 3-chome, Chuo-ku, Sapporo 060-0062, Japan; 7 Research Institute for Advanced Nursing Technology, Osaka Metropolitan University, 1-1 Gakuen-cho, Naka-ku, Sakai, Osaka 599-8531, Japan

**Keywords:** comparative proteomics, proteome analysis, protein language models, ortholog divergence, functional proteomics, proteome complexity, cross-species pharmacology, toxicology, systems biology

## Abstract

Background: Comparative proteome analysis can reveal functional conservation and divergence among orthologous proteins, with important implications for pharmacology and toxicology. Protein language models (PLMs) may capture sequence-derived functional relationships beyond what conventional alignment metrics capture. Methods: Orthologous proteins from *Danio rerio* and *Danio aesculapii* were compared using embeddings generated by the Evolutionary Scale Modeling 2 (ESM-2) protein language model. Reciprocal best-hit inference identified 68,971 high-confidence ortholog pairs, of which 51,086 were available for embedding-based analysis. PLM divergence was quantified using cosine distance and evaluated using length-matched and bitscore-matched random controls, Gene Ontology graph-distance analysis, and localized domain-level comparisons. Results: Ortholog pairs showed strong global conservation, with a median PLM distance of 0.000487, whereas randomized controls exhibited substantially greater divergence. Increasing Gene Ontology graph distance broadened PLM-distance distributions, and leaf–parent comparisons demonstrated significant functional ordering (Wilcoxon *p* = 2.44 × 10^−4^). Local analyses revealed increased divergence in pathophysiologically relevant regions of aryl hydrocarbon receptor (AHR) and potassium channel proteins. Conclusions: PLM embeddings provide a scalable framework for comparative proteome characterization, complement conventional sequence-based analyses, and prioritize orthologs or protein regions with elevated functional divergence for experimental validation in cross-species pharmacology, toxicology, and systems biology.

## 1. Introduction

Comparative proteome analysis provides a framework for understanding how protein conservation and divergence shape biological function across species. As genomic and proteomic datasets continue to expand, there is an increasing need for scalable and reproducible methods that characterize protein similarity beyond conventional pairwise sequence alignment. Recent advances in systems biology, machine learning, and simulation-guided analysis have enabled large-scale modeling of biological systems through workflow automation, representation learning, and reproducible computational strategies [1,2,3,4,5,6]. Reproducibility-focused pipelines are now increasingly applied in molecular modeling, predictive toxicology, and comparative biological inference [7,8,9,10]. These developments support a transition from descriptive sequence comparison toward proteome-scale characterization of functional divergence.

A major challenge in comparative biology, pharmacology, and toxicology is the reliability of cross-species inference. Experimental findings from one organism are often generalized to related taxa, assuming that orthologous proteins retain comparable biological functions. However, receptor-binding kinetics, enzymatic turnover, regulatory signaling, and ligand sensitivity may differ substantially even among closely related species [11]. Species-specific divergence in ligand-binding domains, ion channels, nuclear receptors, and drug-metabolizing enzymes may therefore introduce uncertainty that conventional sequence similarity metrics do not fully capture. Regulatory toxicology frameworks recognize interspecies variability as a major source of uncertainty in hazard assessment and pharmacological prediction [12]. This issue is related to the ortholog conjecture, which proposes that orthologous genes are generally more functionally conserved than paralogs [13]. Nevertheless, high sequence similarity does not always guarantee the conservation of regulatory behavior, ligand response, or the magnitude of phenotypic effect across species.

Traditional comparison methods, including percent identity, BLAST (for example BLAST+ 2.17.0) similarity, and alignment-based scoring, primarily quantify residue-level conservation. Although these approaches remain essential in comparative genomics and proteomics, they do not directly capture distributed contextual dependencies across protein sequence space. Functional similarity is often nonlinear relative to sequence similarity [13], motivating the use of semantic, systems-level, and representation-based approaches. Gene Ontology-based semantic similarity methods provide one strategy for contextualizing biological relationships beyond direct sequence comparison [14,15]. More recently, protein language models (PLMs) trained on large-scale protein sequence corpora have generated embeddings that capture evolutionary constraints, structural regularities, and context-sensitive motif interactions [16,17,18]. Benchmarking studies suggest that PLM-derived embedding distances can approximate functional similarity and identify divergence patterns not readily detectable using alignment-based metrics alone [19,20,21]. These properties make PLM embeddings useful for proteome-scale characterization of ortholog divergence [4,9,22].

An important consideration in proteome analysis is that canonical amino acid sequences represent only one layer of proteome complexity. Functional biological units may also differ through transcript isoforms, alternative splicing, post-translational modifications, proteolytic processing, and proteoform-level variation. Although the present study focuses on canonical protein model sequences, localized divergence within domains, motifs, and ligand-associated regions may provide an initial computational window into functional variation in the proteome. Such analyses can help prioritize proteins and regions for future proteoform-aware or experimentally validated studies.

To evaluate these concepts, this study uses *Danio rerio* and *Danio aesculapii* as a near-neighbor comparative model. *D. rerio* is an established vertebrate model organism widely used in developmental biology, pharmacology, and toxicology because of its rapid embryogenesis, optical transparency, genetic tractability, and compatibility with high-throughput screening systems [23,24]. In contrast, *D. aesculapii* remains comparatively under-characterized despite its close phylogenetic relationship to *D. rerio*. Recent genomic analyses have identified regulatory divergence within the Danio lineage [25]. Because signaling and physiological systems are extensively characterized in *D. rerio* [24,26], the *D. rerio*–*D. aesculapii* comparison provides a controlled framework for evaluating proteome-scale ortholog divergence and its relevance to cross-species pharmacological inference.

This study was thus designed as a proof-of-concept in a closely related species pair, providing a highly conserved proteomic background against which localized divergence can be detected; the resulting distributions should not be generalized to broader evolutionary distances without further validation.

Among the proteins examined, aryl hydrocarbon receptor 2 (AHR2) and Kcnj13 (Kir7.1) provide illustrative examples of localized functional divergence. AHR2 is a ligand-activated transcription factor involved in toxicological responses to environmental contaminants such as dioxins and polycyclic aromatic hydrocarbons [23,26]. Kcnj13 is an inwardly rectifying potassium channel implicated in zebrafish pigment pattern formation [26]. These proteins represent cases in which localized domain or motif divergence may be biologically relevant despite strong overall sequence conservation.

In this study, we evaluated whether PLM-derived embedding distance can serve as a proteome-scale indicator of ortholog divergence between *D. rerio* and *D. aesculapii*. We integrated reciprocal best-hit orthology inference [27], ESM-2-derived cosine distance, local domain-level decomposition, and Gene Ontology-aware statistical analysis [14,28]. We hypothesized that ortholog pairs with larger PLM embedding distances would show greater functional divergence and that localized domain-level analyses would reveal divergence not captured by conventional sequence similarity metrics alone. Rather than treating PLM distance as a direct predictor of pharmacological effect, we use it as a comparative proteome-characterization metric to prioritize orthologs and protein regions for future experimental validation.

## 2. Materials and Methods

### 2.1. Data Source and Analysis Unit

Reference proteomes were obtained from the NCBI RefSeq database for *D. rerio* (assembly GRCz11) and *D. aesculapii* (assembly DanAes1.0). Protein-model accessions were used as the primary analytical units because the objective was comparative characterization of the encoded proteomes rather than gene-level comparison. Quality-control filtering excluded sequences shorter than 50 amino acids, entries containing internal stop codons, and pseudogene-derived models.

Because individual gene loci may generate multiple protein products through alternative transcript usage, the number of protein entries exceeded the number of gene entries. Proteome-scale analyses were therefore conducted at the protein-model level to maximize representation of functional protein diversity. Although the present study focused on canonical protein sequences rather than experimentally resolved proteoforms, inclusion of transcript-derived protein variants provides a broader representation of proteome composition than gene-centric analyses alone. Sequence-quality filtering statistics and ortholog dataset summaries are provided in Table 1.

### 2.2. Ortholog Inference and Candidate Classification

Reciprocal best-hit (RBH) orthologs were inferred using MMseqs2 (v15.6f452) with an E-value threshold of ≤1 × 10^−5^ and a sensitivity parameter of 7.5. Ortholog assignments were constrained using reciprocal query coverage (qcov ≥ 0.8) and sequence length ratio limits of 0.8–1.25. In parallel, DIAMOND (v2.1.8) searches were retained for complementary relationship classification, including one-to-one, one-to-many, many-to-many, and unassigned relationships.

The RBH-constrained set served as the principal high-confidence ortholog dataset for downstream comparative proteome analyses.

### 2.3. Protein Embedding and Distance Calculation

Protein embeddings were generated using the Evolutionary Scale Modeling 2 (ESM-2) protein language model (esm2_t6_8M_UR50D) implemented through the PyTorch 2.8.0 environment. The model produces 320-dimensional sequence embeddings. Final-layer residue embeddings were extracted, and residue mean pooling was applied after excluding special tokens.

This checkpoint was selected because it enabled reproducible, memory-tractable inference across the complete proteome-scale set of orthologs on CPU-based hardware. The objective of this study was to establish a scalable and reproducible workflow for quantifying ortholog divergence, rather than to benchmark protein language model architectures.

Pairwise divergence between orthologs was quantified using cosine distance:

$$d=1-cos\;(e_{1},e_{2})$$

where $e_{1}$ and $e_{2}$ represent pooled embedding vectors for orthologous proteins. Cosine similarity calculations used standard L2 normalization procedures. Embedding inference was performed using CPU-based execution in Python 3.11.

**Figure 1:**
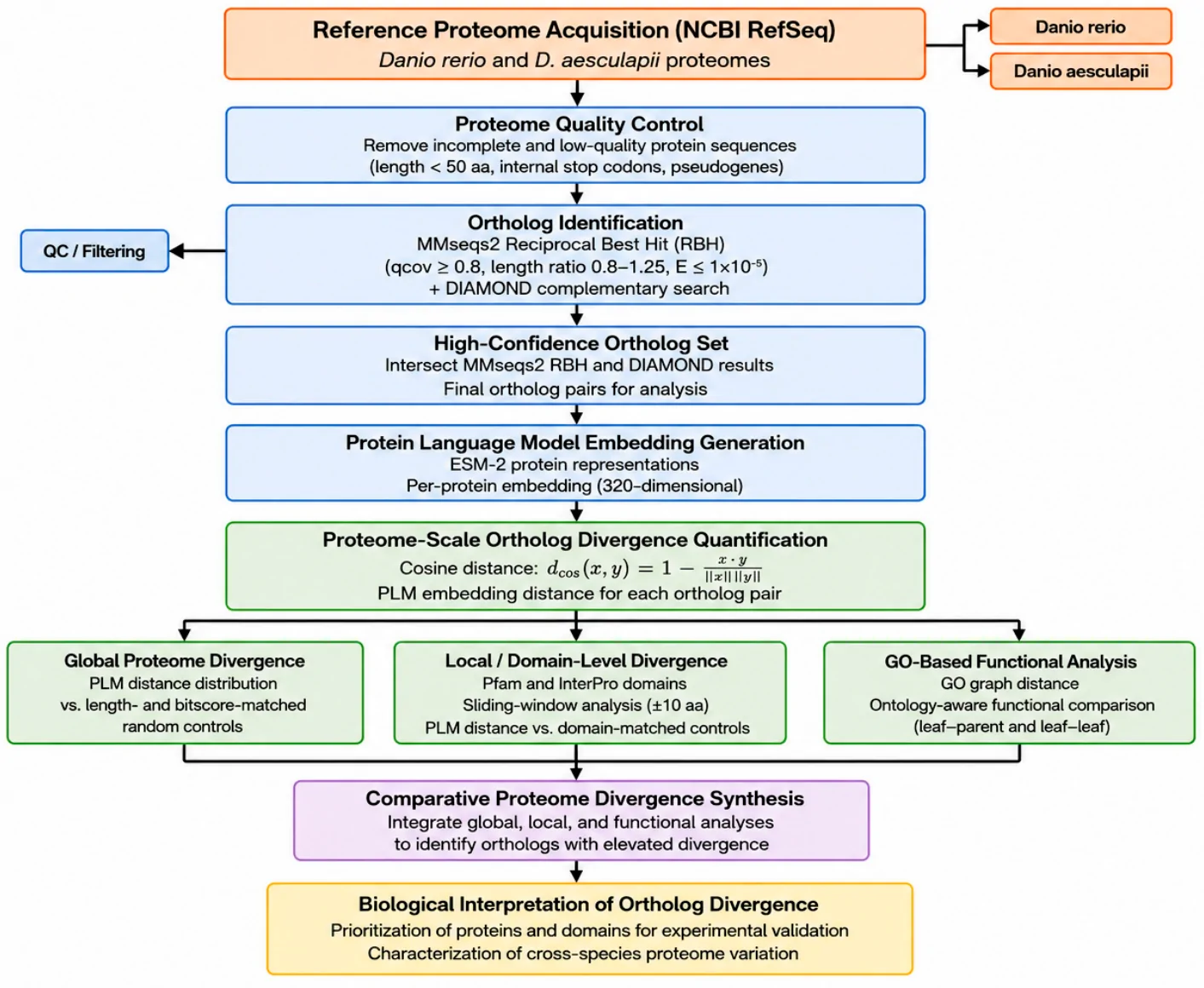
Comparative proteome analysis workflow for ortholog divergence characterization. Reference proteomes of *Danio rerio* and *Danio aesculapii* were filtered, high-confidence orthologs were identified, protein language model embeddings were generated, and PLM-derived cosine distances were analyzed at global, domain-level, and Gene Ontology-based functional levels to characterize cross-species proteome variation.

This embedding-based approach enabled continuous quantification of divergence beyond conventional alignment-based identity metrics. The resulting embeddings provide a proteome-scale representation of protein sequence context that can be used to evaluate functional similarity and divergence among orthologous proteins. The overall computational workflow for ortholog inference, PLM embedding generation, and comparative divergence analysis is summarized in Figure 1.

### 2.4. Proteome-Scale and Localized Divergence Analysis

To distinguish global conservation from localized functional divergence, two complementary analytical strategies were implemented:

1. Domain-level PLM distance analysis using Pfam and InterPro domain partitions;
2. Sliding-window local analysis spanning ±10 amino acids around focal residues or motifs.

This hierarchical decomposition enabled comparison between whole-protein conservation and localized divergence within pharmacologically relevant regions. Local analyses were particularly applied to pigmentation-associated proteins and receptor-associated domains to evaluate whether highly conserved orthologs contained locally amplified divergence signals.

### 2.5. Gene Ontology Distance Analysis

Gene Ontology (GO) shortest-path distances were computed using the go-basic.obo ontology graph. To reduce pseudo-replication arising from exhaustive all-versus-all comparisons, sampling-balanced procedures and controlled resampling strategies were applied.

For hypothesis-concordant testing, leaf molecular-function classes were re-selected under explicit constraints:

- Leaf-class sample size ≥ 50;
- Parent-class sample size ≥ 100;
- Median(leaf) < median(parent).

Ontology-aware comparisons were evaluated using Mann–Whitney U and Wilcoxon statistical procedures. This design enabled controlled assessment of whether increasing GO distance corresponded to broader PLM divergence distributions.

### 2.6. AHR Domain Mapping

AHR2 orthologs (NP_571339 and XP_056303610) were projected onto a shared amino-acid coordinate axis to compare global and localized divergence patterns. Domain regions corresponding to bHLH, PAS-A, PAS-B, and transcriptional activation domains (TADs) were annotated together with the ligand-binding pocket region spanning amino-acid positions 230–350. Segment-specific PLM distances were then mapped onto the corresponding annotated regions to support direct comparison between whole-protein and domain-level divergence.

Domain-level divergence mapping was implemented using the reproducible script reports/scripts/build_Figure 5a_ahr_domain.py which generated reports/fig_ahr_domain_schematic.png and reports/ahr_domain_distance_mapping.tsv.

AHR2 was selected as a representative protein for localized comparative proteome analysis because of its established biological and toxicological relevance.

### 2.7. Statistical Analysis, Reproducibility, and GenAI Use

Statistical analyses were performed in Python 3.11 using NumPy, SciPy, pandas, and matplotlib libraries. Distribution comparisons used nonparametric statistical procedures, including Mann–Whitney U and Wilcoxon tests. Embedding-distance distributions, local divergence analyses, and ontology-aware comparisons were visualized using reproducible plotting workflows.

All tests were two-sided unless otherwise specified. For analyses involving many simultaneous comparisons, particularly Gene Ontology term-level comparisons, *p*-values were adjusted using the Benjamini–Hochberg procedure to control the false discovery rate, and the adjusted values (q-values) are reported. For pre-specified single-hypothesis tests (e.g., the leaf-versus-parent Wilcoxon signed-rank comparison in Section 3.8), no multiple-testing correction was applied, and raw *p*-values are reported.

The computational workflow was implemented using reproducible Python scripts, including:

python reports/scripts/build_Figure 5a_ahr_domain.py

Generated outputs included domain-mapping figures and tabulated segment-level divergence metrics. All workflow steps, including ortholog inference, embedding generation, local decomposition, and statistical analysis, were executed using version-controlled scripts to support reproducibility and auditability of the analytical pipeline.

Generative AI tools were used for language refinement and manuscript organization. No AI systems were used for data generation, statistical analysis, result interpretation, or scientific decision-making. All analyses were performed on publicly available protein sequence datasets, and no experimental proteomic measurements were generated in this study.

## 3. Results

### 3.1. Ortholog Dataset Composition and Denominator Audit

The RBH high-confidence ortholog dataset contained 68,971 constrained one-to-one protein pairs. After integration with available PLM-distance outputs, 51,086 ortholog pairs remained for embedding-based comparison (Table 1). Most excluded pairs were associated with missing *D. aesculapii* identifiers in the DIAMOND candidate universe, while a smaller proportion resulted from unmatched entries during dataset joining.

Coverage statistics for the retained ortholog dataset were as follows: mean query coverage = 0.9901, median = 1.0, minimum = 0.8, and maximum = 1.0.

### 3.2. Global PLM Distribution, Random Controls, and GO Response-Category Overview

**Figure 2:**
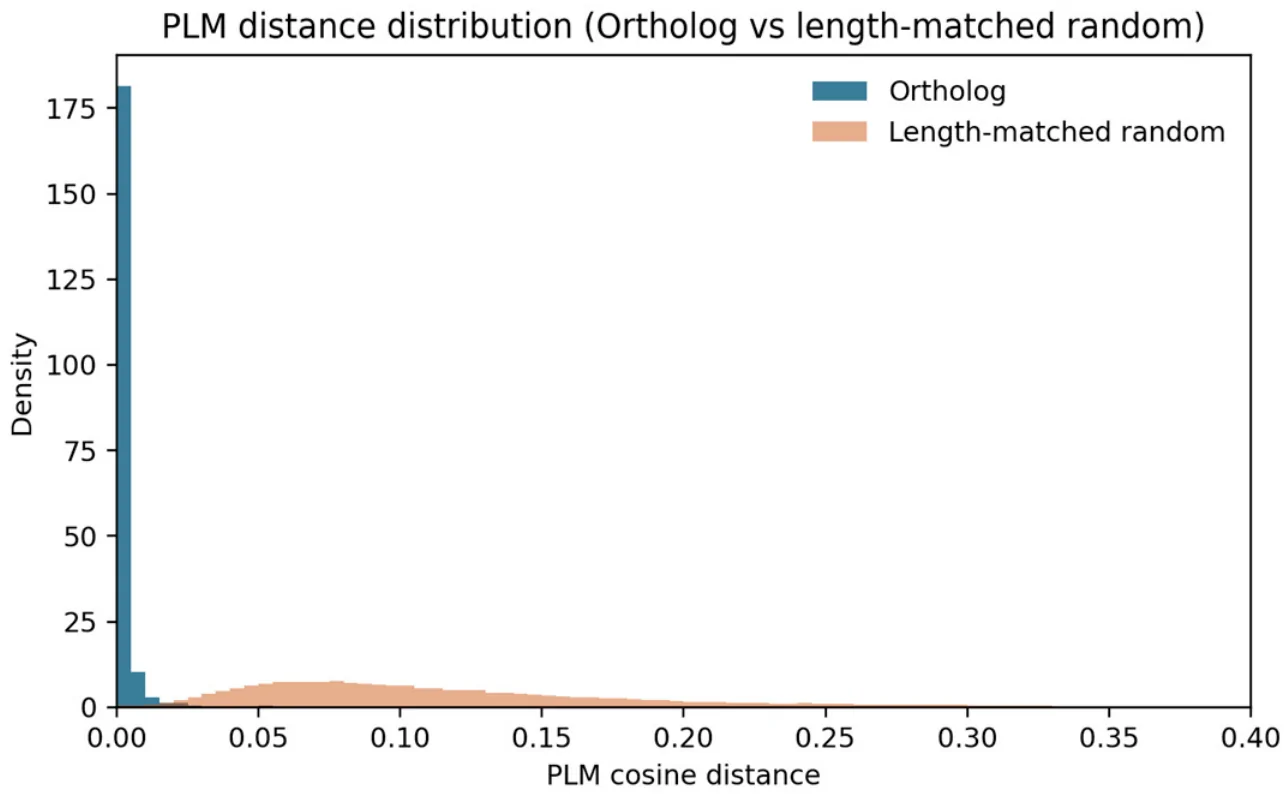
PLM distance distribution. Histogram of PLM distance. X-axis: PLM distance (cosine distance, truncated to 0.4 in plotting). Y-axis: density. Ortholog pairs cluster near zero, while length-matched and bitscore-matched random controls shift toward larger distances.

Ortholog PLM distances were concentrated near zero values, with a median of 0.000487, a 95th percentile of 0.008432, and a 99th percentile of 0.039693. Random-control distributions showed larger PLM distances, with length-matched random pairs (*n* = 200,000) showing a median PLM distance of 0.100962 and bitscore-matched random pairs (*n* = 36,241) showing a median of 0.006505 (Figure 2).

**Figure 3:**
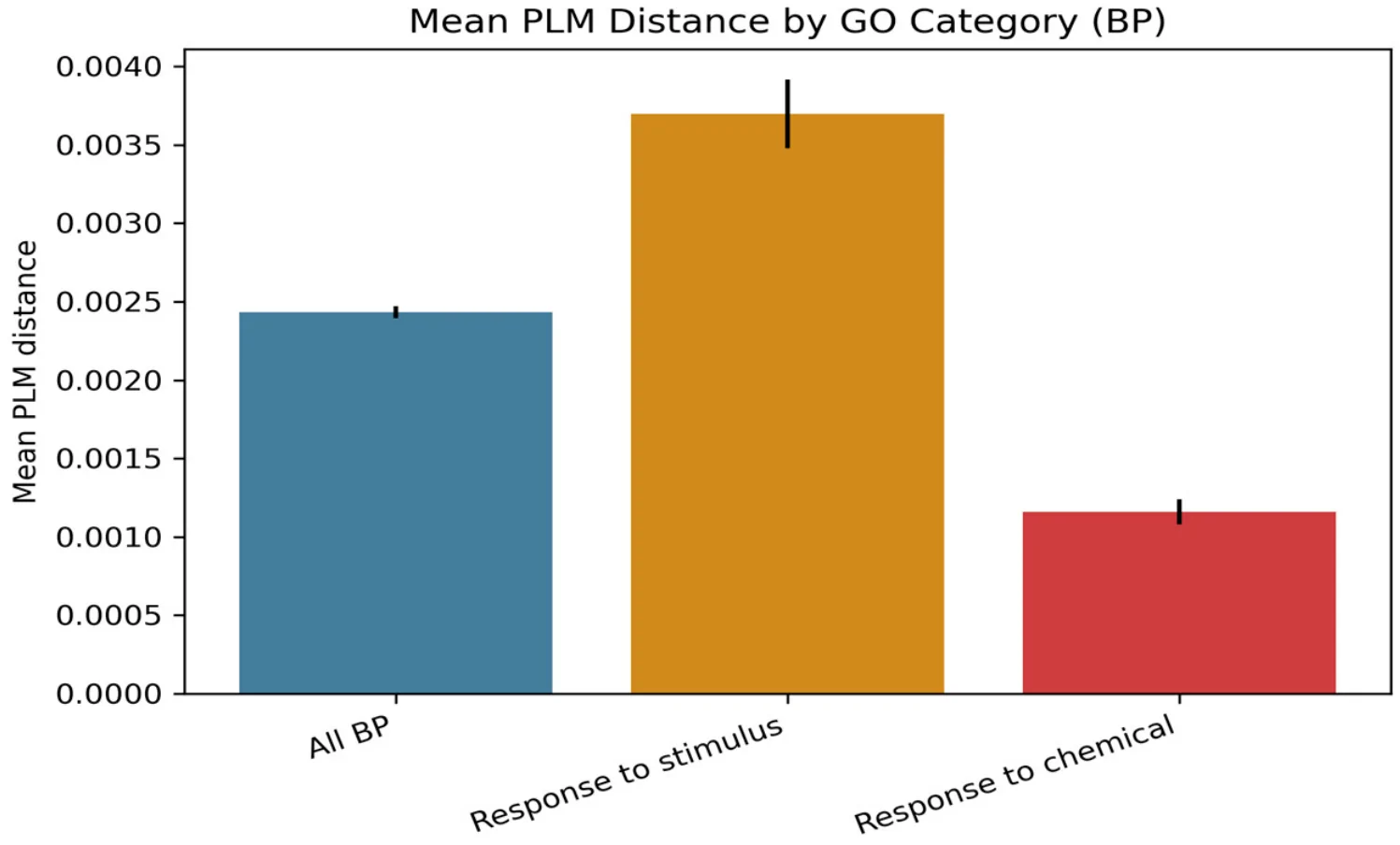
Mean PLM distance by GO response category. Category-level mean PLM distance across response-related GO classes. X-axis: GO response category. Y-axis: mean PLM distance.

Mean PLM distance was compared across selected response-related Gene Ontology biological-process categories. Response-to-stimulus categories showed higher mean PLM distances relative to the overall biological-process background, whereas response-to-chemical categories showed comparatively lower mean PLM distances (Figure 3).

### 3.3. Identity Mismatch Decomposition and PLM Shift

In DIAMOND-based mismatch decomposition, complete-match and partial-mismatch groups showed different PLM-distance distributions in the PLM-joined dataset. Complete-match pairs (*n* = 2819) had a median PLM distance of 0.000000, whereas partial-mismatch pairs (*n* = 48,267) had a median PLM distance of 0.000546.

### 3.4. Localized Divergence in Pigmentation-Associated Proteins

**Figure 4:**
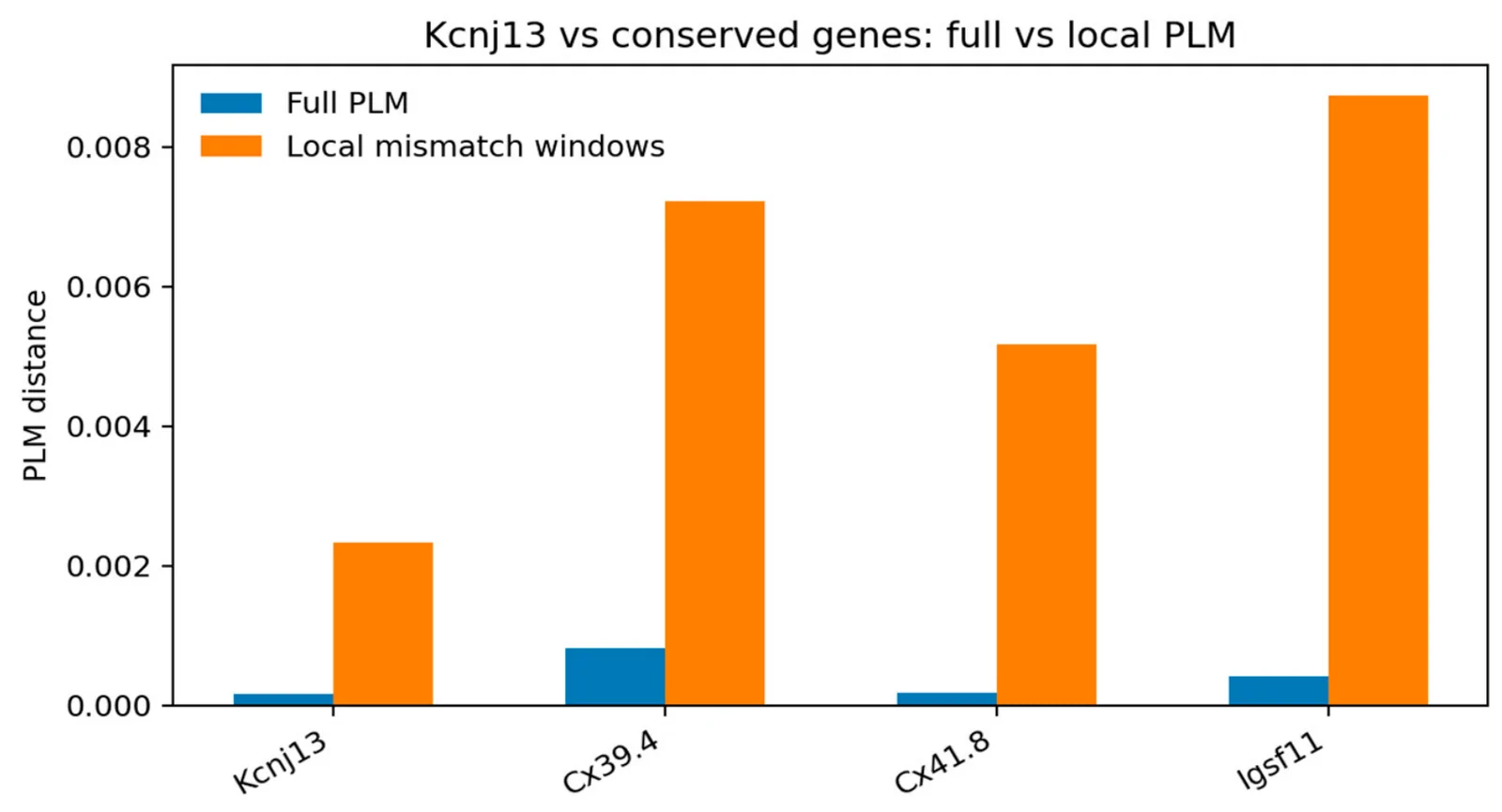
Global vs. local PLM distance in Kcnj13 and conserved pigmentation genes. Bar plot comparing full-length PLM distance and local-window PLM distance. X-axis: target genes. Y-axis: PLM distance.

In *Kcnj13* and other pigmentation-related genes, local PLM distances were larger than full-length distances. *Kcnj13* showed an increase from a full-length PLM distance of 0.000153 to a local mean PLM distance of 0.002338, indicating higher divergence within localized sequence windows despite low global PLM distance (Figure 4).

### 3.5. Bitscore Relationship

**Figure 5:**
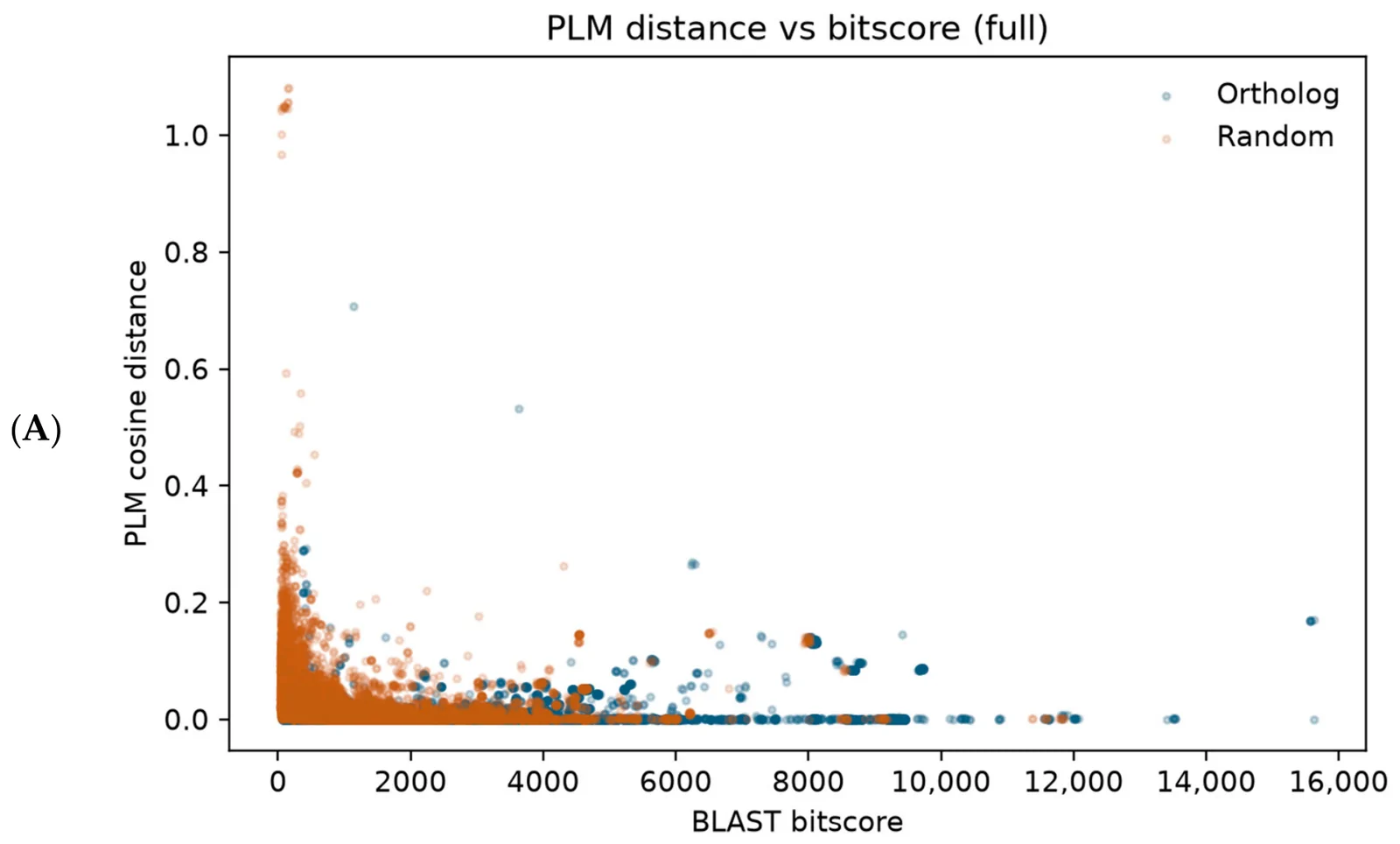

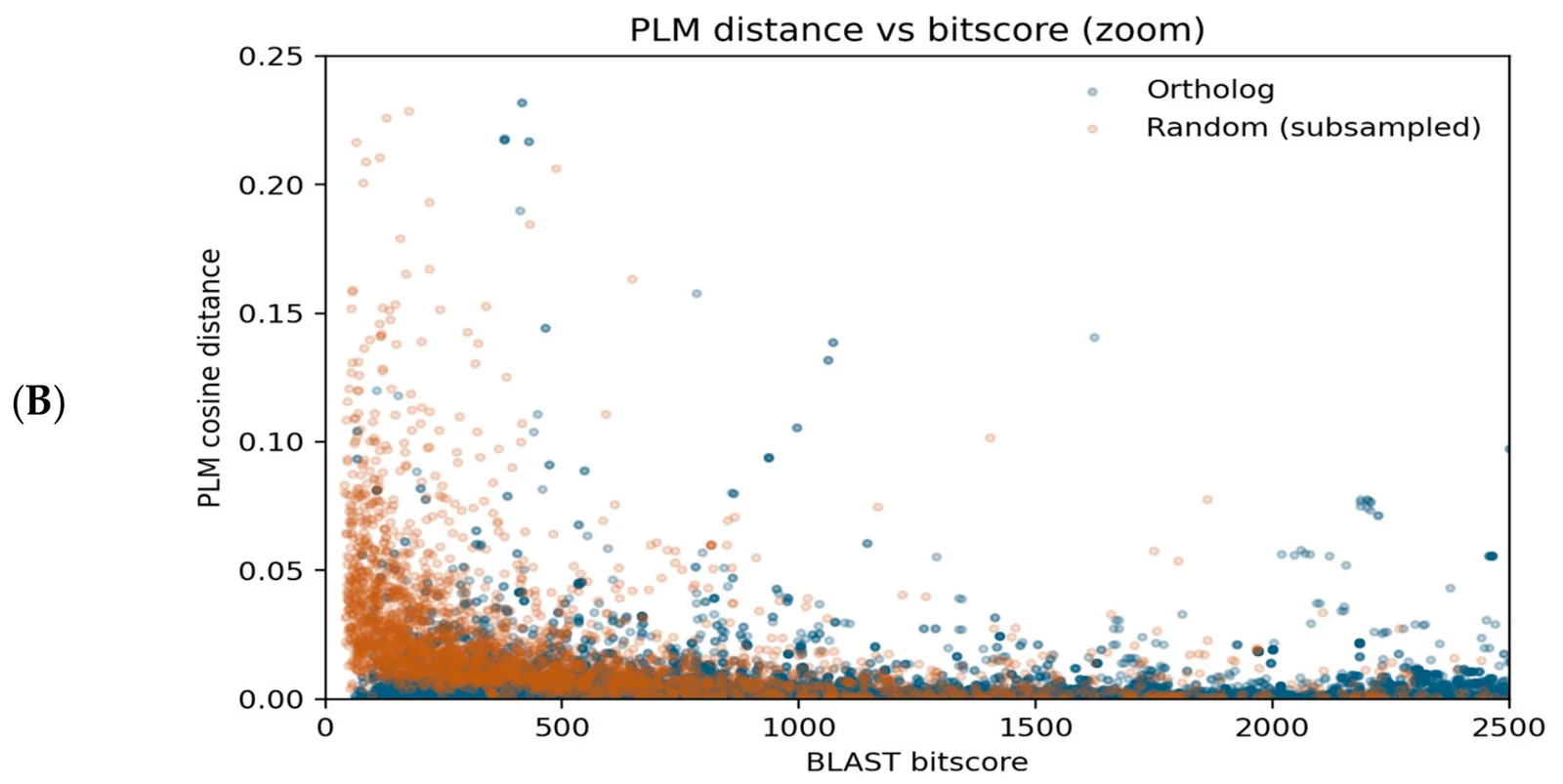
BLAST bitscore vs. PLM distance. (**A**) BLAST bitscore vs. PLM distance (full range). Scatter plot of BLAST bitscore and PLM distance. X-axis: BLAST bitscore. Y-axis: PLM distance. Orthologs and random controls are color-coded. (**B**) BLAST bitscore vs. PLM distance (zoom). Zoomed view of low-bitscore range to improve separation visibility between orthologs and random points.

BLAST bitscore and PLM distance showed similar monotonic trends, with higher bitscore values generally corresponding to smaller PLM distances. However, orthologs and random pairs were not completely separated by bitscore alone, indicating that PLM distance captured partly overlapping but non-redundant information. Random-point thinning was applied to the zoomed low-bitscore range to improve visualization of point separation (Figure 5A,B).

### 3.6. Transcription-Factor and Receptor PLM-Distance Distributions

Transcription-factor and receptor GO categories showed distinct PLM-distance distributions (Table 2). The GO:0003700 transcription-factor activity category (*n* = 2284) showed a median PLM distance of 0.000261 and a 99th percentile of 0.012098, whereas the GO:0004888 transmembrane receptor activity category (*n* = 528) showed a median PLM distance of 0.000353 and a 99th percentile of 0.102650.

**Figure 6:**
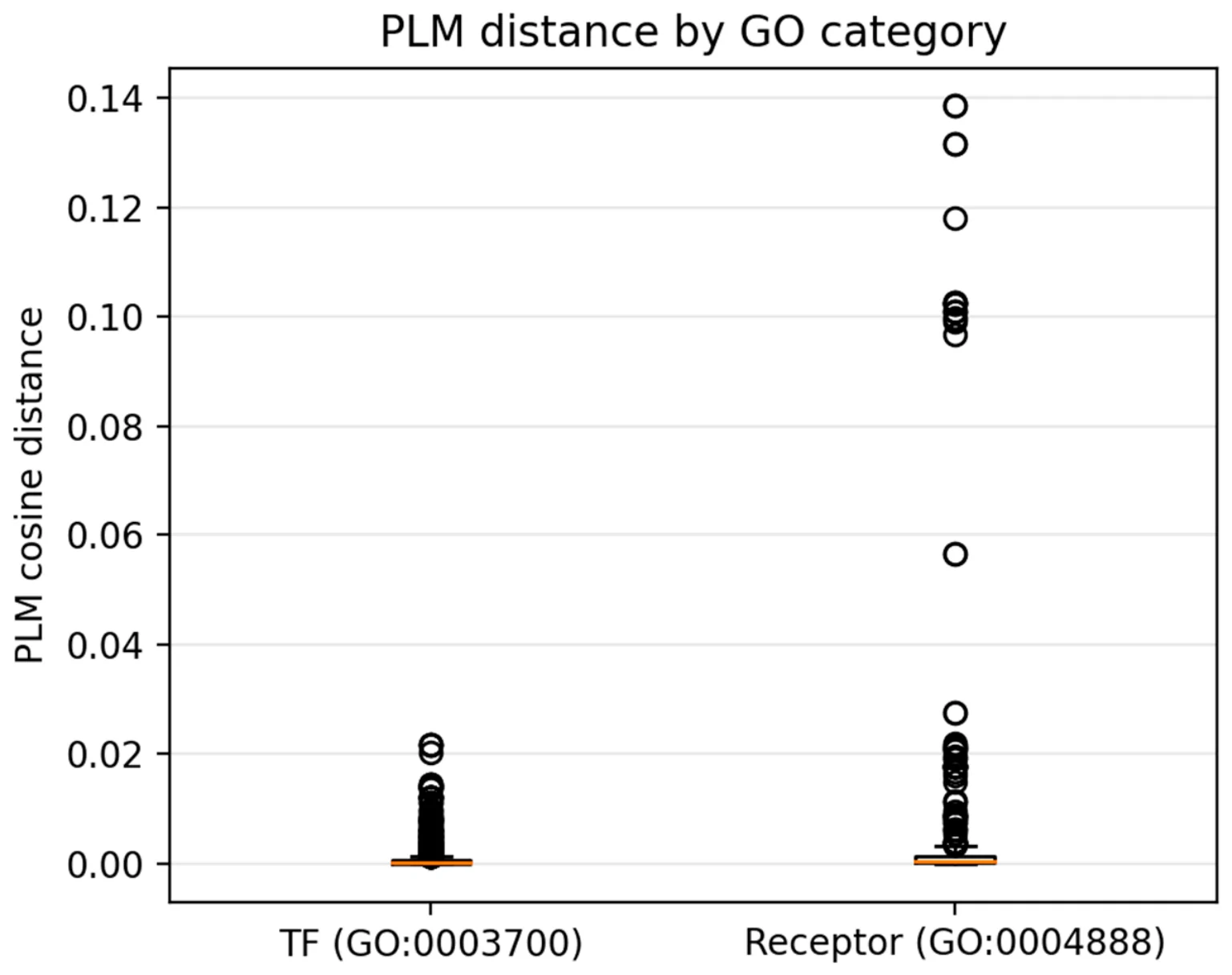
Transcription factor vs. receptor PLM-distance distribution. Boxplot of PLM distances in GO:0003700 (TF activity) and GO:0004888 (transmembrane receptor activity). X-axis: GO category. Y-axis: PLM distance.

These category-level distributions are visualized in Figure 6. Receptor-associated ortholog pairs exhibited broader PLM-distance variation and higher upper-range values than transcription-factor-associated pairs.

### 3.7. GO-Graph Distance Correspondence

**Figure 7:**
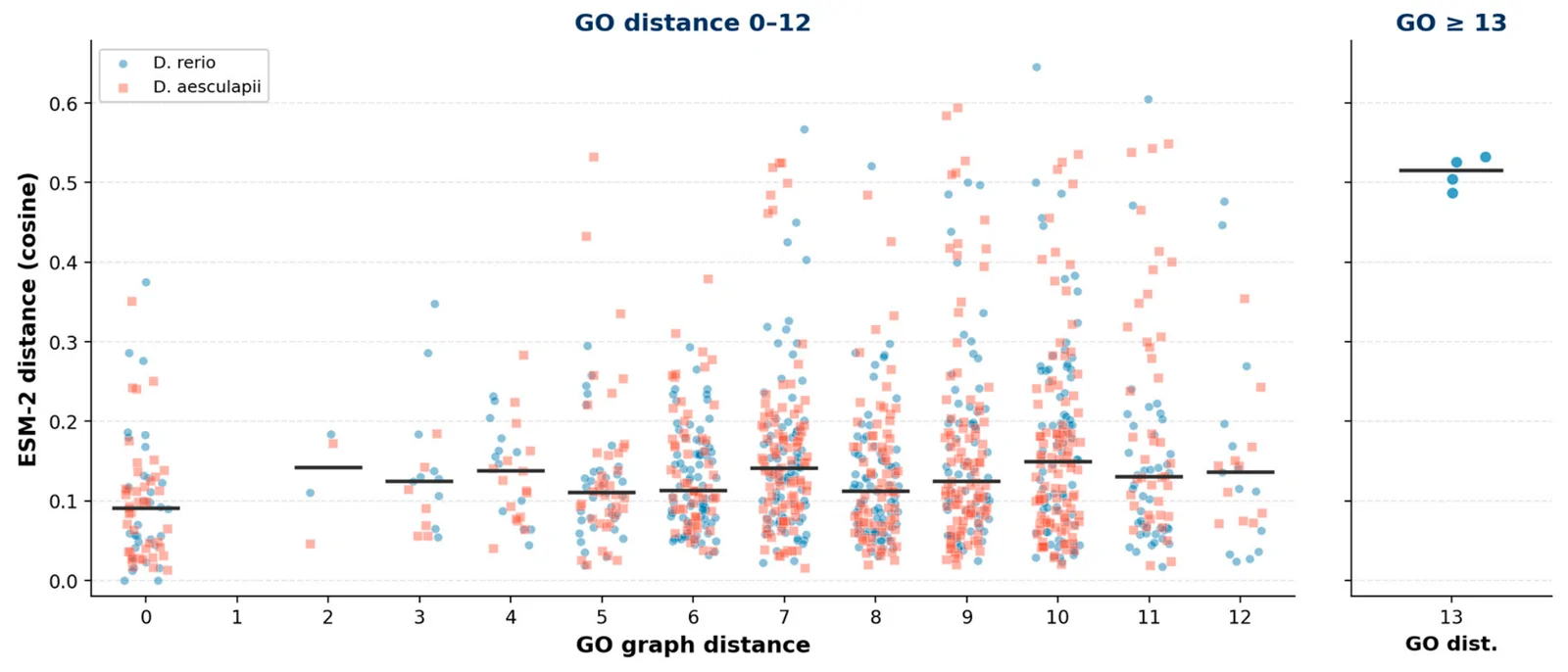
GO-graph distance vs. PLM distance (individual pairs). Scatter plot of GO shortest-path distance (0–13) and ESM-2 distance for orthologous protein pairs. Left panel: GO = 0–12 (*n* = 1103); right panel: GO ≥ 13 (*n* = 4). Blue circles: *D. rerio*; red squares: *D. aesculapii*. Black horizontal bars indicate the median ESM-2 distance within each GO-distance class.

GO-graph distance (0–13) was compared against ESM-2 distance for individual protein pairs (Figure 7). Across the GO = 0–12 range (*n* = 1103), median PLM distances varied modestly across GO-distance classes, although higher GO distances showed broader dispersion toward elevated ESM-2 distances. At GO ≥ 13 (*n* = 4), PLM distances were consistently higher than those observed at lower GO distances. Overall, the results suggest a partial correspondence between GO semantic separation and PLM-based divergence.

### 3.8. Hypothesis-Consistent GO Re-Selection

**Figure 8:**
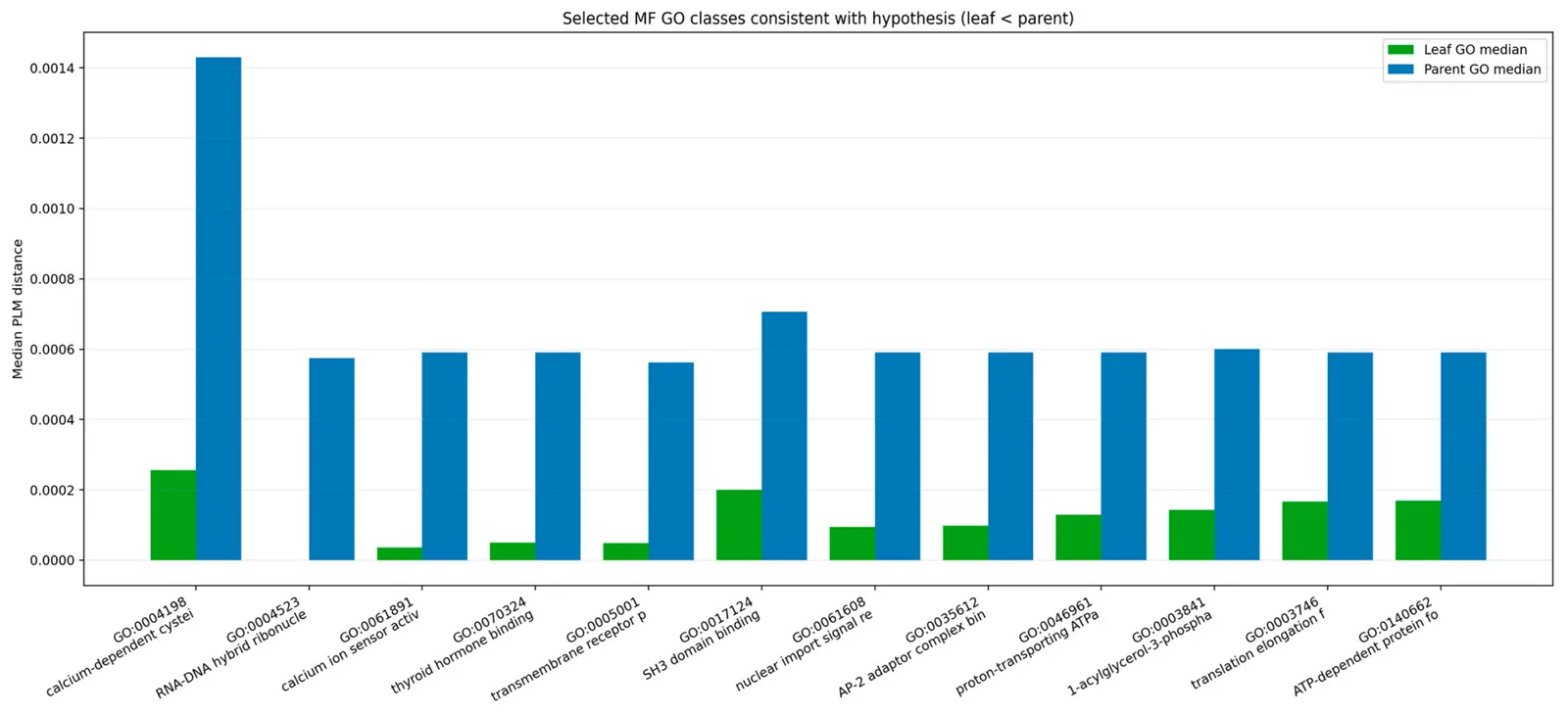
Hypothesis-consistent leaf vs. parent GO classes. Selected molecular-function classes in which leaf GO terms showed lower median PLM distances than their corresponding parent terms. X-axis: selected GO classes. Y-axis: median PLM distance.

To reduce GO-class selection artifacts, leaf molecular-function classes with sufficient sample size and explicit leaf–parent relationships were re-selected. Twelve leaf classes satisfied these criteria. Across the selected classes, median PLM distances for leaf terms were consistently smaller than those of their corresponding parent terms (Figure 8). Pairwise leaf–parent comparison showed a significant shift toward lower leaf-term distances (Wilcoxon signed-rank test, *p* = 2.44 × 10^−4^).

### 3.9. GO:0003700 Parent–Offspring Structure

**Figure 9:**
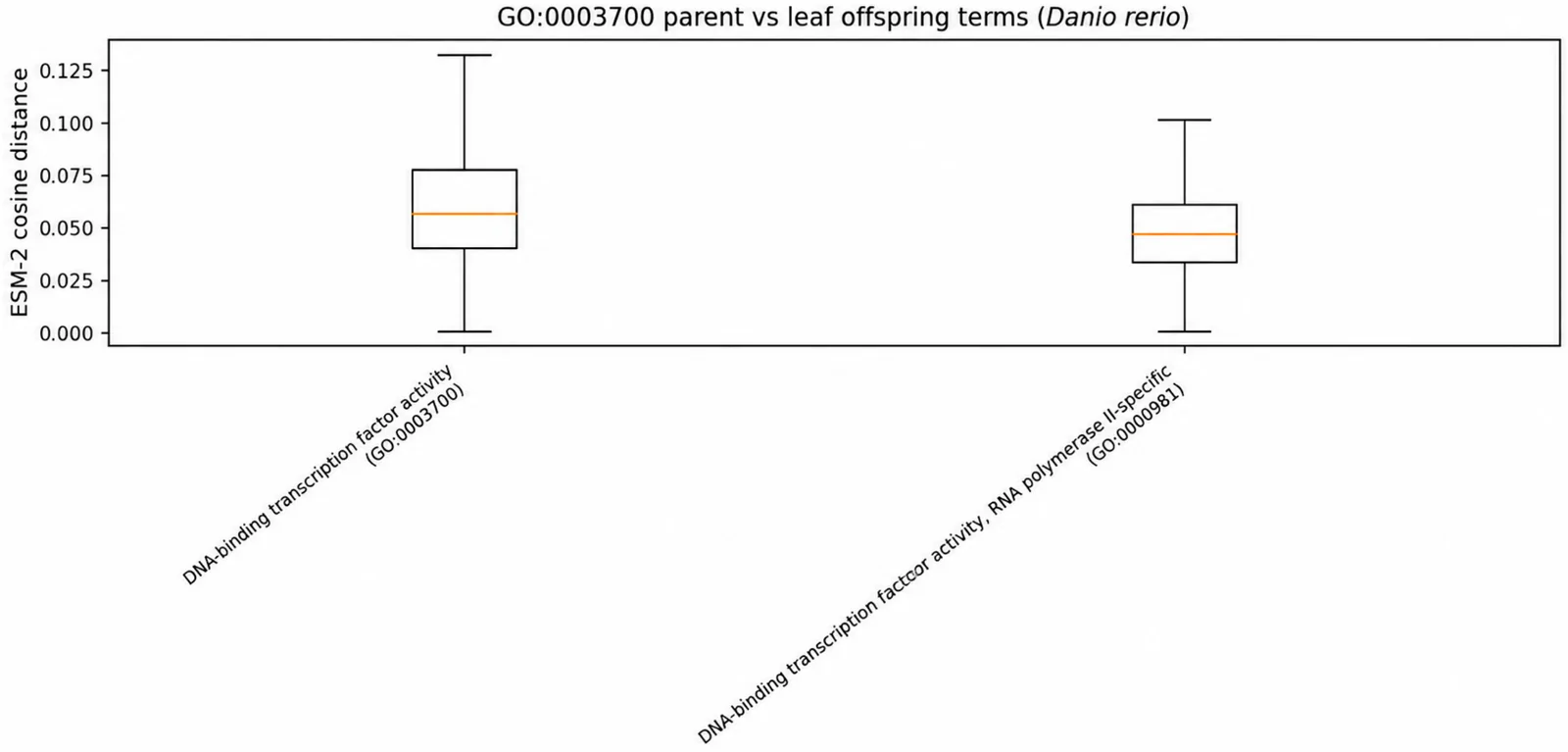
GO:0003700 parent and selected offspring-term PLM distributions. Distribution comparison between GO:0003700 and offspring terms with sufficient sample size. X-axis: GO term. Y-axis: PLM distance.

The GO:0003700 transcription-factor activity category was further compared with selected offspring GO terms showing sufficient representation (Figure 9; Table 3). Distribution comparisons showed variation in PLM distances among parent and offspring categories. Nuclear receptor activity terms exhibited lower median PLM distances, whereas RNA polymerase II-specific transcription-factor subclasses showed comparatively higher median distances. Species-specific sample counts and median PLM distances for selected GO categories are summarized in Table 3.

### 3.10. AHR2 Domain-Level Divergence Mapping

**Figure 10:**
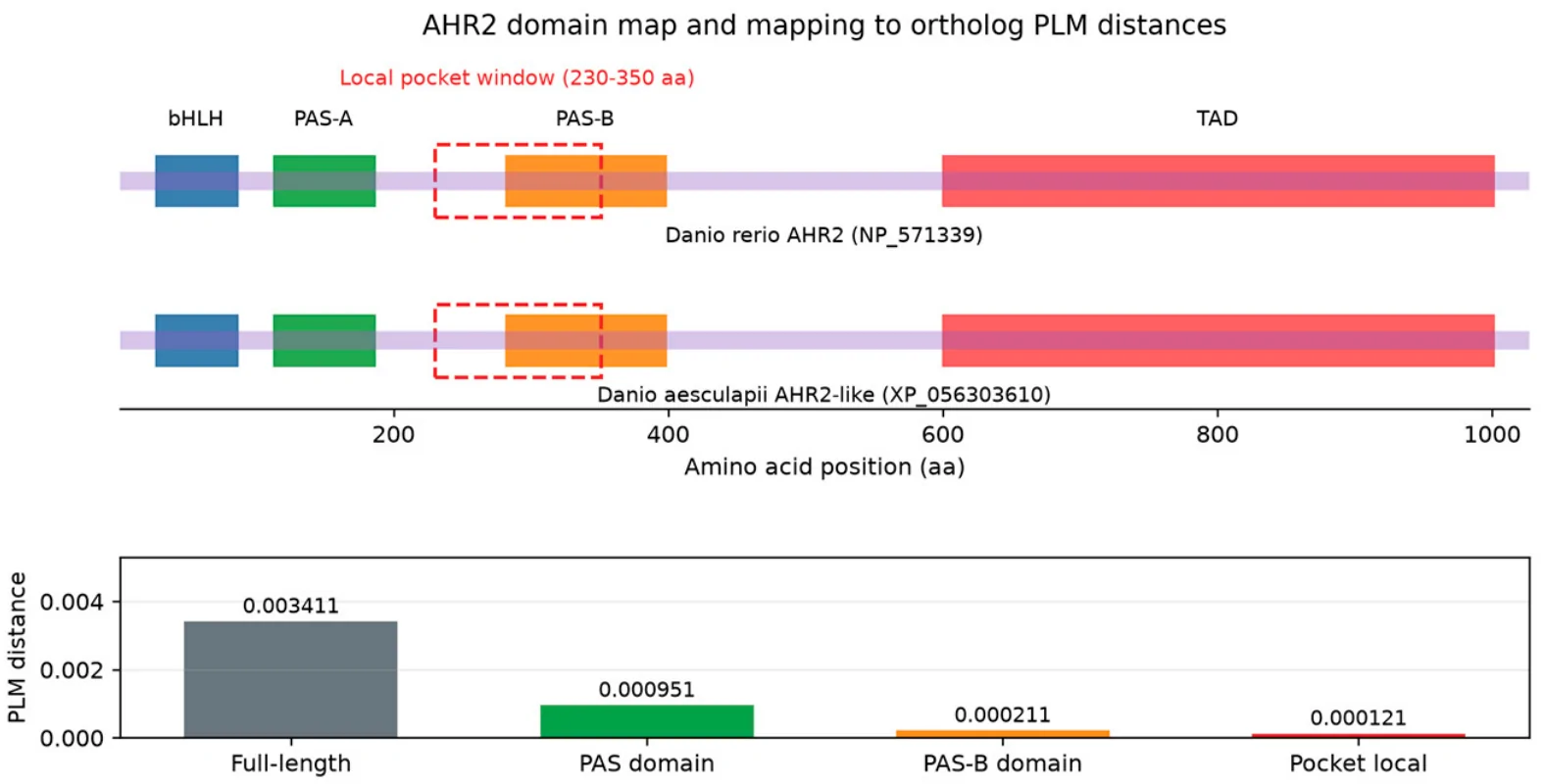
AHR2 domain maps and mapping to ortholog PLM distances. Domain architecture map for AHR2 orthologs and mapped PLM distances for full-length and local segments. Upper panel: amino-acid coordinates with domain blocks. Lower panel: PLM distance by segment.

The AHR2 domain schematic mapped ortholog PLM distances across full-length and segment-level regions. Full-length PLM distance was 0.003411, whereas segment-specific distances were lower in the PAS-A domain (0.000951), PAS-B domain (0.000211), and local pocket region (0.000121) (Figure 10).

### 3.11. AHR Local-Pocket Divergence

**Figure 11:**
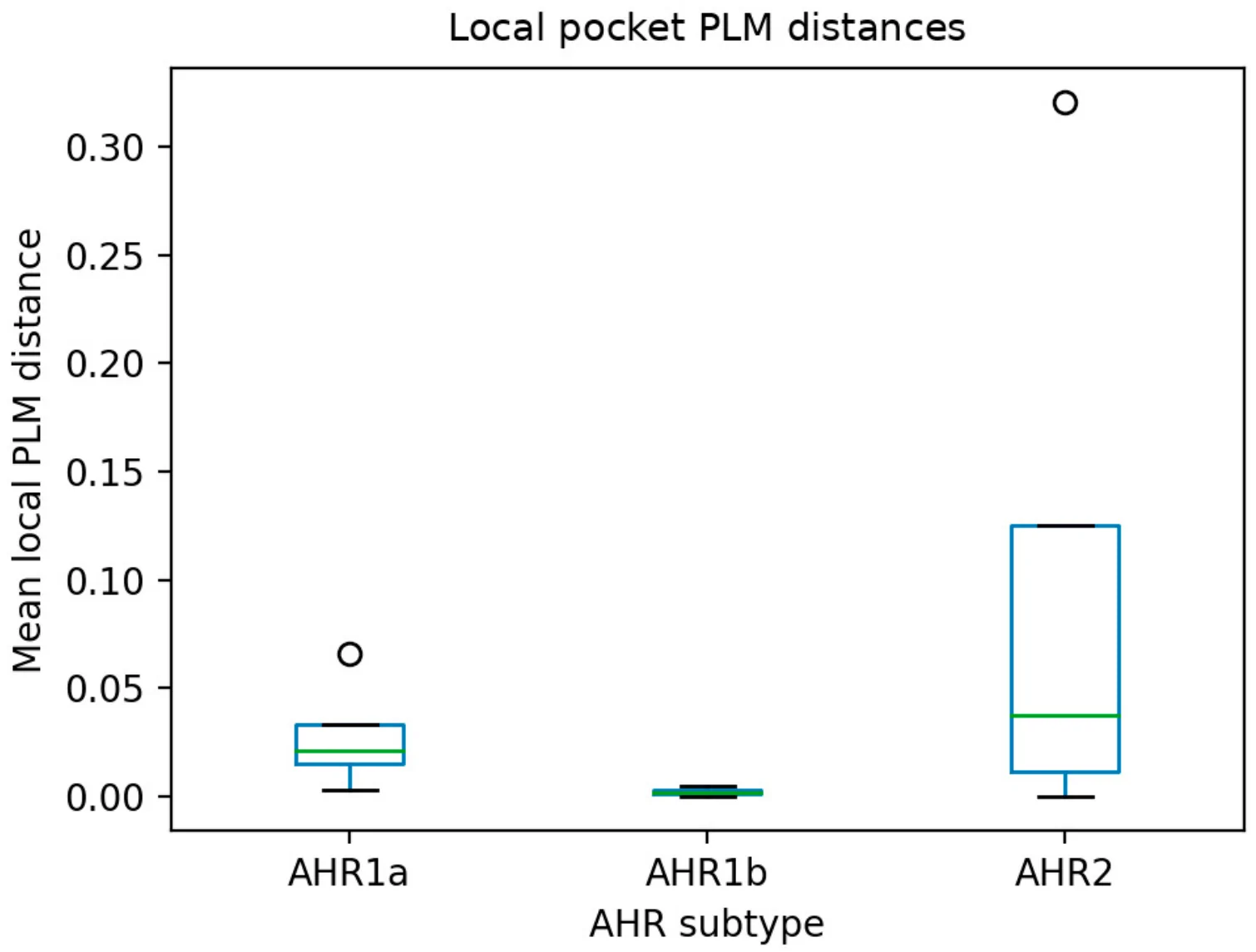
AHR local pocket PLM distance. Distribution of local PLM distances across AHR subtype pocket windows. X-axis: AHR subtype group/window class. Y-axis: local PLM distance. A circle above each box indicates the presence of an outlier, if any.

Local PLM-distance distributions were compared across AHR subtype pocket regions using Takeda-defined residue windows (Figure 11). AHR1a and AHR1b showed relatively low local PLM distances with narrow distributions, whereas AHR2 exhibited broader variation and higher upper-range values. Although median local PLM distances remained low overall, several AHR2-associated regions showed substantially elevated local divergence relative to other subtype groups.

### 3.12. Local Identity and Local PLM-Distance Correspondence

**Figure 12:**
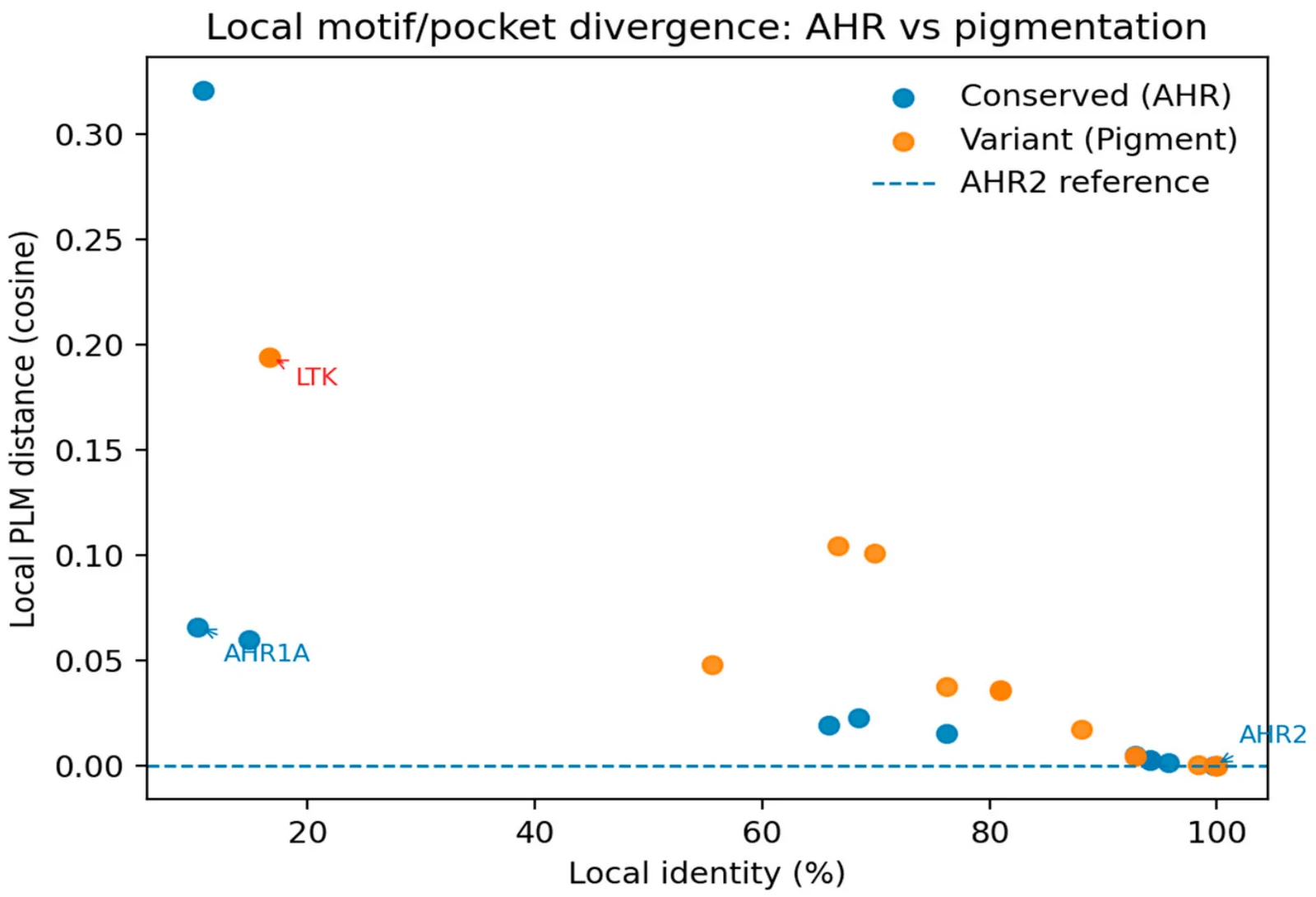
Local identity vs. local PLM distance. Scatter plot comparing local sequence identity and local PLM distance across conserved AHR motifs and pigmentation-related variant regions. X-axis: local identity (%). Y-axis: local PLM distance.

Local identity and local PLM distance were compared across conserved AHR motifs and pigmentation-related variant regions. Conserved AHR regions clustered at high local identity with low PLM distance, whereas pigmentation-related regions showed broader PLM-distance variation across intermediate identity values (Figure 12).

### 3.13. Receptor Subgroup Panel

**Figure 13:**
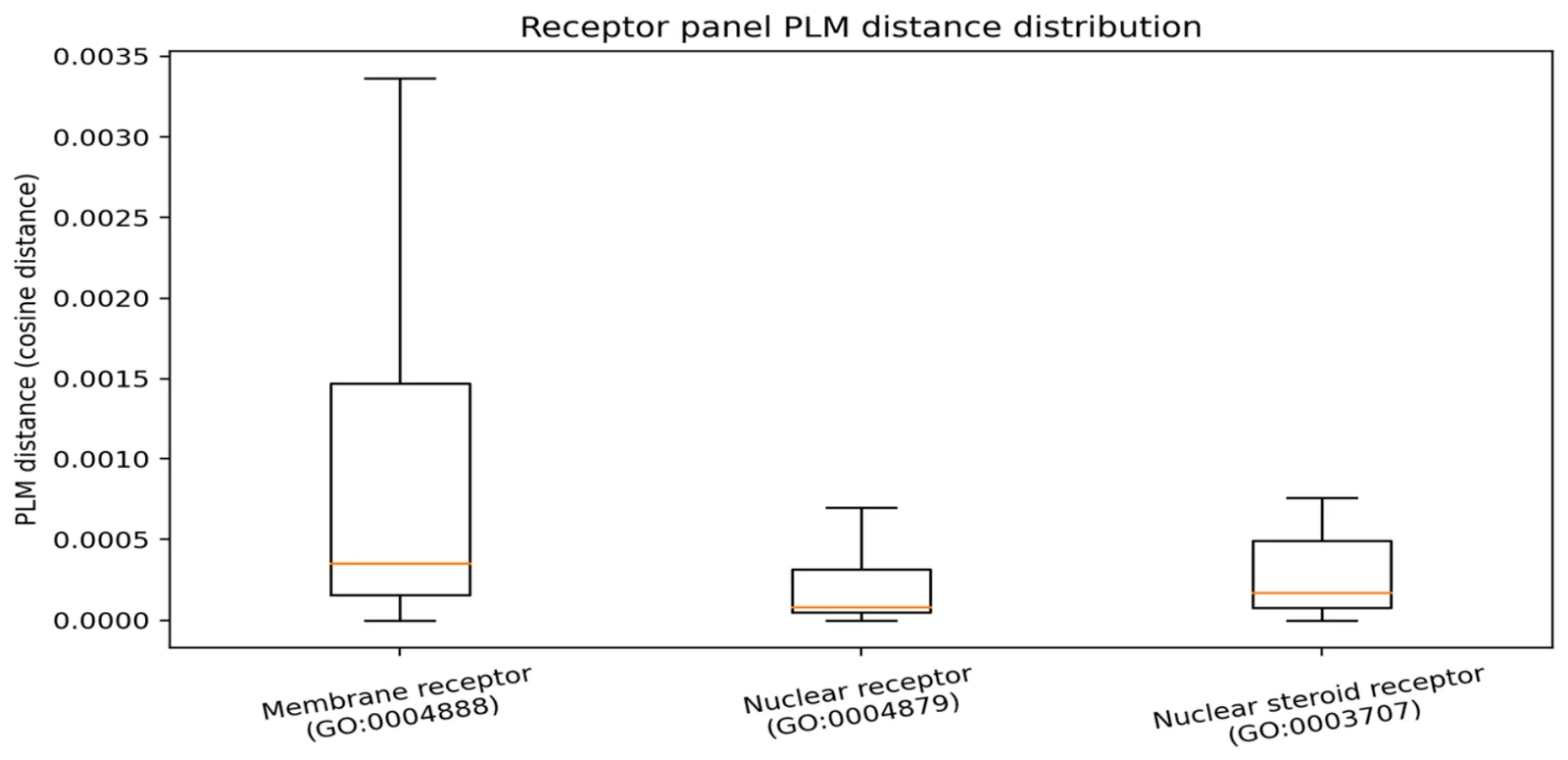
Receptor subgroup local PLM-distance distributions. Distribution of local PLM distances across selected receptor-associated ortholog subclasses. X-axis: receptor subgroup category. Y-axis: local PLM distance.

Receptor-associated ortholog groups showed variable local PLM-distance distributions across selected receptor subclasses (Figure 13). Most receptor groups remained concentrated at low PLM distances, although several subclasses exhibited broader upper-range divergence patterns. Variation among receptor subclasses was more pronounced than that observed for transcription-factor-associated groups, consistent with the broader receptor-category distributions observed in earlier analyses.

## 4. Discussion

This study demonstrates that protein language model (PLM)-derived embeddings can be used for proteome-scale characterization of ortholog divergence between *D. rerio* and *D. aesculapii*. Beyond computational comparison, the approach provides a scalable framework for identifying patterns of functional conservation and divergence that may influence comparative pharmacology and toxicology [11]. Cross-species extrapolation remains a recognized source of uncertainty in toxicological and pharmacological inference, particularly when surrogate organisms are used to predict biological responses in less-characterized species [12]. Although orthologous proteins generally preserve broader functional relationships more reliably than paralogs, orthology itself remains probabilistic rather than absolute [13]. Functionally important divergence may emerge through localized variation within ligand-binding pockets, regulatory motifs, catalytic residues, or domain interfaces despite strong overall sequence conservation. Similar concerns about species-dependent variability in responses have also been emphasized in comparative aquatic toxicology studies [9].

The analytical strategy combined ortholog inference, protein language model embeddings, and ontology-aware functional analysis to characterize proteome-scale ortholog divergence. Ortholog inference followed reciprocal best-hit logic and large-scale sequence-search procedures consistent with established orthology workflows [27]. PLM distance was defined explicitly using reproducible cosine-distance calculations derived from ESM-2 embeddings. In parallel, GO-distance analyses incorporated ontology-aware shortest-path methods grounded in established semantic similarity theory [14,28]. Together, these components establish a transparent computational workflow in which ortholog identification, embedding generation, local decomposition, and ontology comparison remain reproducible at the implementation level.

At the global level, ortholog pairs were clearly separated from both length-matched and bitscore-matched random controls. Ortholog PLM distances remained concentrated near low values, whereas random controls exhibited broader and substantially elevated distributions. Although BLAST bitscore and PLM distance demonstrated similar monotonic behavior, bitscore alone did not fully separate ortholog and random distributions. This observation suggests that embedding-derived metrics capture contextual sequence constraints extending beyond conventional alignment similarity alone. Recent studies on protein language models similarly demonstrate that embedding representations encode structural and functional information not reducible to pairwise sequence identity or alignment score [15,16,17]. Similar embedding-derived continuity across protein functional space has also been observed in recent comparative PLM analyses [6]. Collectively, these findings support the interpretation that PLM embeddings capture latent biological organization across protein sequence space.

The mismatch decomposition analyses further support this interpretation. Increasing mismatch burden corresponded to progressive widening of PLM-distance distributions, indicating that embedding divergence tracks continuous sequence perturbation rather than functioning solely as a binary ortholog classifier. Importantly, however, strong global ortholog conservation did not uniformly predict local conservation. Analyses of pigmentation-associated genes and AHR receptor regions demonstrated that high full-length conservation may coexist with amplified local divergence within functionally important segments. From a pharmacological perspective, this distinction is highly relevant because ligand-binding pockets, PAS domains, gating motifs, and regulatory interfaces may disproportionately influence the magnitude of the response despite otherwise strong global sequence similarity.

The AHR-focused analyses particularly illustrate the utility of localized PLM decomposition. Domain-level and pocket-level analyses revealed substantially reduced divergence within selected PAS and local-pocket regions relative to full-length ortholog comparisons, whereas other subtype-associated regions showed broader divergence distributions. Comparable localized divergence patterns have also been reported in teleost ortholog analyses involving receptor-associated functional regions [4]. These findings align with increasing recognition that localized motif variation and context-dependent structural changes may alter receptor behavior without requiring extensive full-length sequence divergence [18,19]. From a comparative pharmacology standpoint, this observation is important because receptor sensitivity, ligand specificity, and downstream signaling behavior may be influenced more strongly by restricted functional regions than by overall ortholog conservation alone.

The GO-based analyses require careful interpretation. Broad transcription-factor and receptor categories showed distinct PLM-distance distributions, with receptor-associated groups exhibiting broader divergence at the upper end than transcription-factor-associated groups. Similarly, the higher PLM distances observed among response-to-stimulus categories may reflect greater evolutionary flexibility among proteins involved in environmental sensing and physiological adaptation, whereas the lower distances observed in response-to-chemical categories may indicate stronger conservation among orthologs involved in chemical-response pathways. At the ontology level, moderate GO-distance categories did not consistently separate from identical-function bins under unrestricted sampling. However, when GO leaf classes were re-selected under explicit granularity and minimum sample-size constraints, the expected leaf-versus-parent ordering emerged with statistical support. These observations are consistent with previous studies demonstrating that functional similarity within Gene Ontology depends strongly on semantic granularity, graph topology, and annotation structure [29,30]. The findings therefore suggest that ontology-aware filtering is important when interpreting embedding-based functional-distance relationships.

The present study also highlights the importance of transparent data integration and annotation-aware analysis in comparative proteomics. Large-scale embedding analyses can become difficult to interpret when ortholog definitions, filtering criteria, or annotation-selection procedures are incompletely documented. By maintaining explicit accounting across ortholog inference, mismatch decomposition, GO filtering, and local-region analyses, the study improves interpretability and reproducibility of comparative proteome analyses. Such transparency is particularly important when biological conclusions are derived from large-scale computational characterization of protein repertoires.

The predictions obtained in this experiment will need to be verified through functional analysis in the future. Several limitations should also be acknowledged. First, PLM distance remains an indirect proxy for biological divergence rather than a direct quantitative measure of the magnitude of pharmacological effect. Embedding similarity alone cannot independently establish receptor activity, ligand affinity, signaling outcome, or toxicological response without experimental validation. Second, GO annotations remain incomplete and unevenly distributed across functional classes, which may influence ontology-level comparisons despite balanced sampling procedures. Third, the present analyses focused on pairwise ortholog comparisons between *D. rerio* and *D. aesculapii* and therefore do not yet capture broader phylogenetic scaling behavior across multiple teleost lineages. Finally, local-window analyses remain sensitive to region selection and annotation quality, particularly for poorly characterized motifs or domains.

In addition, the embedding distances reported here are conditional on the selected ESM-2 checkpoint and may vary with model architecture, parameter size, and training corpus; comparison across larger ESM-2 variants and alternative protein language models is an important direction for evaluating the robustness and generalizability of the present framework.

Although mean pooling yields an efficient whole-protein representation suitable for proteome-scale comparison, it can attenuate highly localized signals, particularly in long or multidomain proteins. To mitigate this, whole-protein analysis was complemented by domain-level and sliding-window local analyses (Section 2.4 and Section 3.4, and Section 3.10, Section 3.11, Section 3.12 and Section 3.13); systematic evaluation of alternative pooling strategies and sequence-length effects remains future work.

A further consideration concerns the level of proteome representation. Our framework operates at the canonical protein level, treating each RefSeq protein model accession as a single representative sequence per gene locus. While transcript isoforms are partially captured, this approach does not enumerate the broader landscape of proteoforms—distinct molecular species generated through alternative splicing, alternative transcription start sites, sequence variation, post-translational modifications, and proteolytic processing—that collectively constitute the functional proteome of each species. Cross-species divergence at the proteoform level may amplify or modulate the canonical-sequence uncertainty quantified here, particularly because pharmacological and toxicological responses can depend strongly on tissue- and condition-specific proteoform expression. Likewise, protein species (sensu stricto) within a single proteoform may diverge in post-translational modification signatures, even when their primary sequences are identical, adding a further dimension of proteome complexity that is not directly addressed in the present framework. Extending the present PLM-based approach toward proteoform-aware comparisons—through joint embedding of splice-variant ensembles, integration of PTM-resolved sequence annotations, or coupling with mass-spectrometry-derived proteoform inventories—represents a natural next step for capturing proteome complexity in cross-species pharmacological inference.

Although protein language models encode aspects of structural organization, embedding distance should not be interpreted as equivalent to structural alignment; integration of AlphaFold-predicted or experimentally determined structures will provide complementary validation of the localized divergence reported here.

Despite these limitations, PLM-derived embeddings provide a scalable and biologically interpretable approach for comparative proteome characterization and prioritization of orthologs exhibiting elevated functional divergence. PLM distance should therefore be interpreted primarily as a triage-oriented comparative metric rather than a direct predictor of pharmacological effect size. Similar uncertainty-aware prioritization approaches have also been proposed for comparative toxicological and pharmacological screening workflows [10]. In practical applications, embedding-based divergence screening may help guide rational allocation of experimental validation resources by identifying orthologs, domains, or local motifs that warrant closer functional investigation. Future work may extend this framework toward larger comparative datasets, experimentally validated receptor systems, multi-species phylogenetic scaling, integration with structural prediction models or ligand-interaction simulations, and proteoform-aware comparative proteome analysis.

## 5. Conclusions

This study shows that PLM-derived distance can provide a scalable and biologically interpretable measure of ortholog divergence between *D. rerio* and *D. aesculapii.* Orthologs showed low global PLM distances, but local analyses revealed amplified divergence in selected functional regions, including pigmentation-associated proteins and AHR receptor domains. These findings highlight an important principle: strong global ortholog conservation does not always guarantee local functional conservation.

By combining ortholog inference, PLM embeddings, GO-aware analysis, and local-domain decomposition, this approach offers a practical strategy for identifying protein pairs or regions with elevated functional divergence. PLM distance should not be treated as a direct predictor of pharmacological effect but rather as a prioritization metric to guide targeted experimental validation in comparative pharmacology, toxicology, and comparative proteomics.

## Figures and Tables

**Table 1:** Set definitions and denominator audit.

Set	Definition	Count
RBH high-confidence 1:1 set	MMseqs2 RBH constrained by qcov and length ratio	68,971
DIAMOND broad candidate set	Top 90% bitscore candidates retained	88,309
PLM-joined set	Pairs with available PLM distance in joined table	51,086

**Table 2:** PLM-distance summary by functional group.

Category	*n*	Mean	Median	95th Percentile	99th Percentile
GO:0003700 (DNA-binding TF activity)	2284	0.000795	0.000261	0.002964	0.012098
GO:0004888 (transmembrane receptor activity)	528	0.005725	0.000353	0.020895	0.102650
All orthologs	68,971	0.002432	0.000487	0.008432	0.039693

Note: GO:0004872 had no usable entries in this dataset and was not used as the receptor-group label.

**Table 3:** GO:0003700 parent–offspring summary (species-wise).

GO ID	Term	*n* (*rerio*)	Median (*rerio*)	*n* (*aesculapii*)	Median (*aesculapii*)
GO:0004879	Nuclear receptor activity	194	0.017313	116	0.018407
GO:0003707	Nuclear steroid receptor activity	58	0.019338	40	0.018174
GO:0001227	DNA-binding transcription repressor activity, RNA polymerase II-specific	161	0.046763	90	0.055227
GO:0003700	DNA-binding transcription factor activity	300	0.056946	300	0.061195
GO:0001228	DNA-binding transcription activator activity, RNA polymerase II-specific	152	0.065618	100	0.064610
GO:0000981	DNA-binding transcription factor activity, RNA polymerase II-specific	300	0.067127	300	0.061809

## Data Availability

All original contributions of this study are presented within the article. Further questions may be addressed to the corresponding authors.
